# Editorial: Break the mental health stigma: the role of emotional intelligence

**DOI:** 10.3389/fpsyt.2024.1386289

**Published:** 2024-02-23

**Authors:** Carlos Laranjeira, Sigita Lesinskiene

**Affiliations:** ^1^ School of Health Sciences, Polytechnic of Leiria, Leiria, Portugal; ^2^ Centre for Innovative Care and Health Technology (ciTechCare), Polytechnic of Leiria, Leiria, Portugal; ^3^ Comprehensive Health Research Centre (CHRC), University of Évora, Évora, Portugal; ^4^ Clinic of Psychiatry, Institute of Clinical Medicine, Faculty of Medicine, Vilnius University, Vilnius, Lithuania

**Keywords:** mental health stigma, emotional intelligence, life span, stigma, awareness

The Research Topic “*Break the mental health stigma: the role of emotional intelligence*” endeavours to contribute to the understanding of potential links between emotional intelligence (EI) and mental health stigma. Research findings will inform policymaking, public health, and clinical best practices. Understanding emotions is a fundamental complement to emotional competence, which according to theoretical assumptions is a predictor of social adjustment and mental health. This leads to a positive association between high levels of EI and mental health (1). Emotions are fundamental in the daily lives of individuals, aiding in decision-making and adaptive responses to various adversities. They also play an important role in helping to preserve social well-being and emotional/subjective well-being. Research during the COVID-19 pandemic demonstrated that online EI training is effective at sustaining critical aspects of mental health during a subsequent real-life crisis (2). Knowing how to manage emotions allows individuals to identify their affective states and adjust their reactions, action, thought, and behaviour to adaptively deal with emotional experiences. An individual’s ability to express, self-regulate, and understand emotions is called emotional competence. Conversely, suppressing or denying emotions negatively influences mental health (3). As both constructs, emotions, and well-being, are related, people with a high tendency to suppress/deny emotions tend to present low levels of subjective well-being. EI also plays a significant role in the development of psychosomatic symptoms and disorders when somatic health is related to emotional regulation and general mental health.

The evidence provides a well-documented connection between EI and psychopathologies including depression, stress, and anxiety (4). These conditions are characterized by negative attributions, unpleasant cognitions, and self-stigma. An elevated degree of EI can safeguard against depressive situations by enhancing the recognition of emotions, identifying unpleasant moods, and fostering overall life contentment (1, 5). Research also suggests that individuals with strong emotional abilities are less susceptible to mental health issues, leading to an improved quality of life, well-being, and enhanced sense of belonging (5, 6). EI is a beneficial psychological trait that facilitates the establishment of a balance between challenging life circumstances and the ability to adjust. Conversely, there is a correlation between low levels of EI and the emergence of depressive and anxiety symptoms (7).

Research shows that EI capacities and coping styles are of utmost importance in family divorce when building mutual parenting is complicated and full of personal and interpersonal challenges (8, 9). Individuals affected by parental divorce have a higher risk of developing a variety of mental health conditions, and psychosomatic and psychosocial disorders. A meta-analysis shows a consistent direction of influence regarding the long-term effect of parental divorce on their children, and further research should focus on developing programs to promote the resilience of children affected by divorce (10). Recent research also demonstrated that adolescents’ mental health problems increase after parental divorce, these effects are long-lasting and underline the need for better care for children with divorcing parents (11).

The literature suggests that the relationship between EI and mental health is due to the perception that a more emotionally intelligent individual is more aware of their emotions and better able to regulate them. Therefore, they present higher levels of well-being, a stronger and richer social network, and more effective coping strategies, which reduce levels of internalized and public stigma (12). Stigma is associated with multiple adverse health outcomes, including (but not limited to) delayed treatment initiation, avoidance of clinical interactions, inadequate treatment adherence, psychological distress, mental illness, and a greater probability of recurrence of health issues (13–16). Mental health services have many stigma-related factors needed to design and implement interventions at the individual (patient, staff) and structural level (in health policy and the environment) (17). The health literature has acknowledged the difficulties and repercussions of stigma for some time, but there is still considerable uncertainty regarding the personal experience of those living with stigma. To decrease the stigma associated with mental illness and promote emotional regulation, positive mental health literacy should be considered an outcome of mental health promotion actions and a mediator between mental health and well-being, allowing the person to prevent the development of mental disorders (18). One of the most sensitive and vulnerable groups is children of parents with mental illness (COPMI), they need complex sources for support and strengthening EI. Decreasing stigma and raising awareness of society about sensitive COPMI topics and the complex needs of these families is also of utmost importance and still underdeveloped (17). Children and adolescents who grow up with foetal alcohol spectrum disorders also face stigma and long-term educational, medical, and social difficulties; the importance of finding relative ways to enhance EI is little researched (19).

The present six academic papers focus on different target groups. Three of them are from China (n=3), and there is a single article from Colombia, Turkey, and the United States of America. They appear in distinct formats, including opinion papers, review papers, and empirical papers employing diverse research designs (including cross-sectional and longitudinal studies). Below is a concise overview of their novel contributions and recurring themes. Xu et al., examine “the associations among mental health related eHealth literacy (eHL), mental health-seeking attitude, and wellbeing among Chinese young electronic media users during the COVID-19 pandemic” (p.1). Bu et al., examine “the associations between attitudes toward seeking professional psychological help, self-stigmatization of seeking help, Perceived Social Support, and optimism in a cohort of Chinese high-school students” (p.1). Wu et al., explore “the self-processing characteristics of individuals with social anxiety disorder from the first-person perspective and the third-person perspective” (p.1). Hernández et al., perform a narrative review “to evaluate the epidemiology, associated factors, quality of evidence and propose possible strategies for the control of suicide in physicians” (p.1). In a longitudinal case-controlled study, Uyar and Donmezdil compared Turkish healthcare and non-healthcare workers in terms of obsessive-compulsive and depressive symptoms. Finally, in an opinion piece, Melita attempts “to summarize the neurobiological and physiological effects of emotional labour on attorneys, as well as provide several means of counteracting them” (p.1).

Understanding the fundamental emotional, cognitive, and social processes and the applied and clinical situations can help unify and expand our knowledge of EI and mental health stigma throughout life, as demonstrated by the papers on this Research Topic. However, the research herein is not extensive; consequently, the results must be evaluated considering the numerous methodological and conceptual limitations cited in the publications. Little researched areas remain on EI’s importance in situations when people face significant life challenges such as divorces, complicated grief, mental illness, and crises. Nevertheless, it is our conviction that by providing a comprehensive outline of the subject matter and placing particular emphasis on the latest developments, this Research Topic will prove beneficial to individuals with varying levels of expertise and contribute toward better mental health for everyone worldwide.

## Author contributions

CL: Writing – original draft, Writing – review & editing. SL: Writing – original draft, Writing – review & editing.
